# Early life predictors of adolescent suicidal thoughts and adverse outcomes in two population-based cohort studies

**DOI:** 10.1371/journal.pone.0183182

**Published:** 2017-08-10

**Authors:** Jennifer Dykxhoorn, Simon Hatcher, Marie-Hélène Roy-Gagnon, Ian Colman

**Affiliations:** 1 School of Epidemiology & Public Health, Faculty of Medicine, University of Ottawa, Ottawa, Ontario Canada; 2 Division of Psychiatry, Faculty of Brain Sciences, University College London, London, United Kingdom; 3 Department of Psychiatry, Faculty of Medicine, University of Ottawa, Ottawa, Ontario, Canada; Yokohama City University, JAPAN

## Abstract

**Background:**

Understanding suicidality has proven challenging given the complex aetiology in early childhood. Being able to accurately predict groups at increased risk of developing suicidal thoughts may aid in the development of targeted prevention programs that mitigate increased vulnerability. Further, the predictors of suicidal thoughts may be shared with other outcomes in adolescence. Previous research has linked many factors to suicidality, so the objective of this study was to consider how these factors may act together to increase risk of suicidal thoughts and other non-mental health outcomes.

**Methods:**

Two longitudinal datasets were used in this analysis: the *National Longitudinal Survey of Children and Youth* (NLSCY) and the *Avon Longitudinal Survey of Parents and Children* (ALSPAC). A Classification and Regression Tree model comprised of 75 factors describing early childhood was constructed to identify subgroups of adolescents at high risk of suicidal thoughts in the NLSCY and was validated in ALSPAC. These subgroups were investigated to see if they also had elevated rates of antisocial behaviour, substance misuse, poor physical health, poor mental health, risky health behaviours, and/or poor academic performance.

**Results:**

The sensitivity was calculated to be 22·7%, specificity was 89·2%, positive predictive value 17·8%, and negative predictive value 91·8% and had similar accuracy in the validation dataset. The models were better at predicting other adverse outcomes compared to suicidal thoughts.

**Conclusion:**

There are groups of risk factors present in early life that can predict higher risk of suicidality in adolescence. Notably, these factors were also predictive of a range of adverse outcomes in adolescence.

## Introduction

Suicide is the second leading cause of death in 15–29 year-olds worldwide [1]. As most people who die by suicide have previously thought about the act [2], suicidal thoughts are useful markers of later suicidal behaviours and deaths. Suicidal thoughts are an indicator of significant distress, and have been associated with a range of psychological difficulties and negative health states including: poor psychosocial functioning; risky sexual behaviour; aggressive behaviour; substance use disorders; and, depressive disorders [3,4].

Understanding and predicting suicidal thoughts, behaviours, and deaths has proven challenging given the complex aetiology beginning early in the life course [3,5,6]. There is a robust literature on suicide risk factors and research has identified a wide range of factors at the individual [3,7–10], family [11–13], and neighbourhood levels [14–16] to increased suicidality. However, much previous research have considered risk factors in isolation and predictive power for suicidal outcomes has been low despite over five decades of research attempting to better understand suicidality [17]. Being able to accurately predict groups of children and youth at increased risk of developing suicidal thoughts may aid in the development of targeted prevention programs that mitigate increased vulnerability. Where much of the research on suicide prediction have focused on factors present near the time of suicidal thoughts and behaviours, a better understanding of the early life factors may inform early prevention strategies [18]. Many risk factors for suicidal thoughts are markers of early-childhood difficulty, and thus may also be able to predict other adverse outcomes in adolescence. Research shows that there is a close inter-relationship between psychological, and behavioural problems, which tend to co-occur and stem from the same conditions [19]. Thus, predictors of suicidal thoughts may also shed light on the development of other important adolescent outcomes [20]. Consequently, early-life intervention programs for groups at high risk of adolescent suicidal thoughts have the potential to enhance many domains of health and social behaviours. The robust risk factor literature on suicide has clearly established the existence of numerous risk factors for suicide; however, these have largely been investigated individually.

### Aims of the study

The aim of this study was to assess the contribution of the interactions between biological, psychosocial, social, and environmental factors present in early childhood that predict high risk of suicidal thoughts. Further, this research aimed to investigate if there are shared predictors between suicidal thoughts and other negative health and social outcomes in adolescence.

## Methods

### Study design

**Fig 1:**
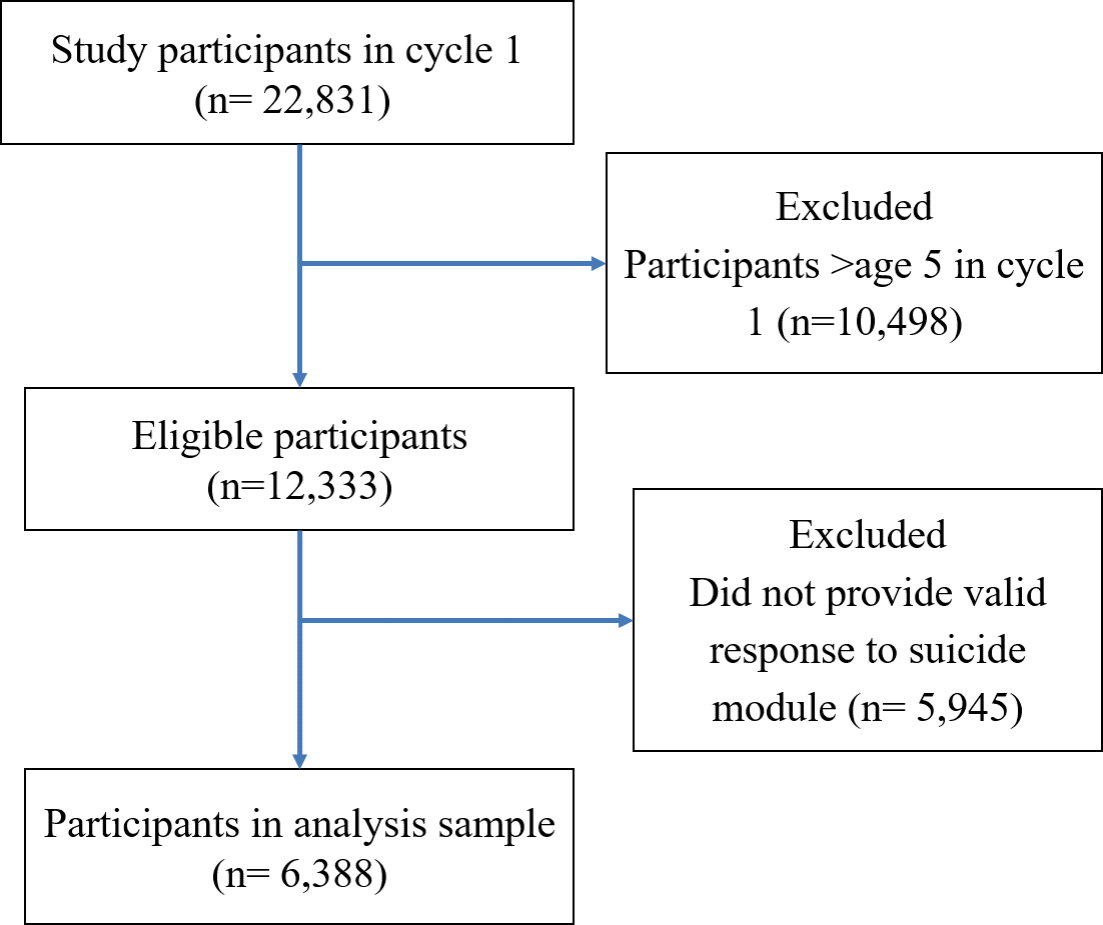
Study population and analysis cohort, NLSCY CONSORT flow diagram, Canada, 1994–2009.

Two longitudinal datasets were used in this analysis: the *National Longitudinal Survey of Children and Youth* (NLSCY) and the *Avon Longitudinal Survey of Parents and Children* (ALSPAC). The NLSCY is a long-term study of Canadian children followed from childhood to early adulthood [21]. It started in 1994/1995 and collected information biennially on health, physical development, social environment, and general well-being until 2008/2009 [21]. More detailed information on the study available at www.statcan.gc.ca/imdb-bmdi/4450-eng.htm. For this study, participants over the age of five in cycle one were excluded, as were those who did not have at least one valid response to the suicidal thoughts question at age 12 or older (Fig 1).

**Fig 2:**
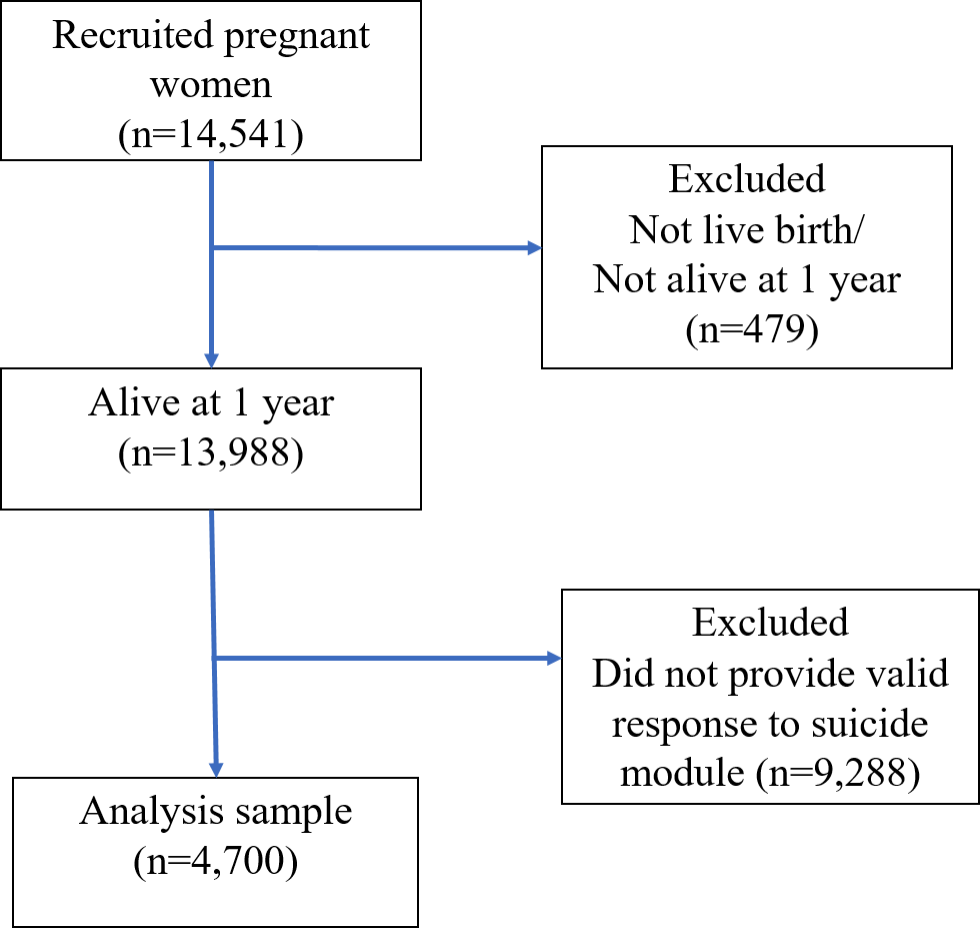
Study population and analysis cohort, ALSPAC CONSORT flow diagram, United Kingdom, 1991–2008.

Five years of age was selected as the cut-off for assessing early life exposures, as this is the age at which most Canadian children enter school. The unweighted sample size for this study was 6,388. The Avon Longitudinal Study of Parents and Children (ALSPAC), was also used. ALSPAC follows a large population cohort of children from early life into adolescence and beyond, collecting extensive details on childhood health and development [22]. This analysis was based on participants whose mothers were recruited in 1991–1992 (n = 14,541) resulting in live births (n = 14,062), who were alive at one year of age (n = 13,988), and who had provided a response to the suicide module, for a final analysis sample size of 4,700 (Fig 2) [22].

Please note that the study website contains details of all the data that is available through a fully searchable data dictionary: <http://www.bristol.ac.uk/alspac/researchers/data-access/data-dictionary/> [23].

### Primary outcome

In the NLSCY, study participants aged 12 or older were asked “In the past 12 months, did you *seriously* consider attempting suicide?” (‘yes’ = 1, ‘no’ = 0). In ALSPAC, suicidal thoughts were assessed with the question “Have you **ever** thought of killing yourself, even if you would not really do it?” Those who answered ‘yes’ were asked about the timing of these thoughts, with those reporting thoughts in the past 12 months coded as ‘yes’.

### Secondary outcomes

Six secondary outcomes were considered which covered a range of other self-reported adverse health and social outcomes in adolescence. These included antisocial behaviour, substance misuse, poor physical health, poor mental health, participation in risky health behaviours, and poor academic performance. Full details on the secondary outcomes can be found in S1–S7 Tables. Antisocial behaviour was measured using a 9-item risky behaviour questionnaire, where depending on the severity of the item, participants were coded as exhibiting antisocial behaviour if they reported any of the behaviours in the past 12 months either at least once (e.g. fought with someone to the point where they needed to care for their injuries’), or for the less severe items, the participant was consider to show antisocial behaviour if it was reported three or more times (e.g., ‘intentionally damaged or destroyed anything that didn’t belong to you’) (S1 Table). Similarly, different cut-offs were used for substance use if they endorsed one or more or the following drug use patterns: cigarettes or alcohol (daily or weekly), marijuana (once or twice a month or more frequently), or misusing illegal/prescription drugs (ever) (S2 Table). Poor physical health was determined based on the frequency of common physical health complaints (including headaches and/or stomach aches and/or backaches) and assessment of their general health (‘fair’ or ‘poor’) (S3 Table). Mental health was assessed using the shortened Centre for Epidemiologic Studies Depression Scale, which is a 12-item scale that participants will score between 0 and 36. Those with a score of nine or above were said to have symptoms consistent with the clinical diagnosis of depression (S4 Table) [24]. Similarly, anxiety was assessed using the Anxiety and Emotional Disorders Scale, a seven-item scale where participants could score between 0–14, where a score of eight or more was considered to indicate a moderate or high level of anxiety (S5 Table) [25]. Participants were said to have poor mental health if they had depression and/or anxiety. Several items were used to categorize participants as participating in risky health behaviours, including ‘operated a motor vehicle after drinking or doing drugs’ (‘3 or 4 times’ or ‘5 times or more’), sexual intercourse without a condom, or ‘seldom/never’ wearing a seat belt when riding in a car (S6 Table). Finally, participants were classified as having poor academic outcomes if they felt they were doing ‘poorly’ in their school work or if they reported: ‘skipped a day of school without permission’ (‘3 or 4 times’, ‘5 times or more’), ‘been suspended from school’ (‘once or twice’, ‘3 or 4 times’, ‘5 times or more’), ‘dropped out of school’ (‘yes’) (S7 Table).

### Predictor variables

Utilizing a conceptual model incorporating aspects of the social ecological model with the suicide continuum [26,27], predictor variables were selected, based on a systematic literature review (S1 Fig). 75 risk and protective factors for adolescent suicidality were included, covering pre- and post-natal, individual, familial, interpersonal, and environmental domains. Predictive factors included information collected from the mother and the children focusing on information collected at ages four and five as well as important information from early life that could be recalled in a screening situation. All predictors were dichotomized and individuals were not excluded due to item non-response. See S8 Table for further details on the predictor variables.

### Statistical analysis

A Classification and Regression Tree (CART) model was built to predict suicidal thoughts at age 12 or older using NLSCY data. CART models have the capacity to model non-parametric, high-order interactions through recursively portioning the sample in order to identify the best combinations of predictor variables that identify sub-groups of adolescents that differ in terms of risk of suicidal thoughts. Predictor variables were dichotomized and those with item non-response for a predictor were kept in the model (1) yes or (2) no/missing and progressed to the next split in the tree. The minimum node size (n_min_) was set at 64 individuals, representing 1% of the overall sample [28,29]. In maximal tree, terminal nodes were assessed as ‘high’, ‘moderate’, or ‘low’ risk of suicidal thoughts: nodes with greater than twice the base prevalence rate of suicidal thoughts were designated as ‘high risk’; nodes with less than half the base rate were deemed ‘low risk’; and, nodes with prevalence between these two cut-offs were said to have ‘moderate’ risk [30].

Branches of the tree were pruned from the maximal model so that if the subsequent splits did not change the designation of the nodes between these three risk levels, the tree was pruned to the parent node after which no further differentiation in level of risk occurred. For example, if the parent node was designated as “low risk” and all the splits following that node continued to differentiate the child nodes all at “low risk” as well, the tree was pruned to the parent node. Based on the final tree results, the sensitivity, specificity, positive predictive value, and negative predictive value was calculated based on high-risk vs. not high-risk, as classified by the models. A multivariate logistic regression model, using stepwise selection was also built to compare the CART model to more traditional modelling.

In order to validate the model, data from the ALSPAC was used. Study participants who were alive at one year and who later provided a response to the suicide module in adolescence were included in the analysis. The 35 predictors which had determined the splits in the NLSCY model were utilized, selecting the measure and time point which most closely replicated the measure available in the NLSCY (S9 Table). Participants in ALSPAC were classified as “high risk” according to the model developed in the NLSCY and sensitivity, specificity, positive predictive value, and negative predictive value were calculated. The high-risk classification generated to predict suicidal thoughts in the NLSCY was also used to assess the secondary outcomes in the NLSCY cohort.

For analysis in the NLSCY, longitudinal weights were used. The longitudinal weighting strategy from Statistics Canada was based on a series of cascaded adjustments, where the initial weight was adjusted for non-response and post-stratification to generate the longitudinal weights. This analysis was completed using SAS version 9·3 and R version 3·0·3, including the RPART and LOGISTIC packages. Ethical approval for the study was obtained from the Ottawa Health Science Network Research Ethics Board, the ALSPAC Ethics and Law Committee, and the Local Research Ethics Committees.

## Results

The weighted prevalence of reported suicidal ideation was 12·0% in the NLSCY sample of 6,388. Suicidal thoughts were found to vary according to most predictive variables in bivariate analysis (Table 1).

**Table 1:** Predictive factors—demographic and health characteristics, NLSCY, n = 6,388, Canada, 1994–2009.

	Time of data collection	N (un-weighted)	% (weighted)	% (weighted) with suicidal thoughts
Gender	Study enrolment			
Girl		3,190	49.5	15.6
Boy		3,198	50.5	8.5
Prenatal problems	Cycle 1			
Yes–at least one		741	8.0	11.9
No problems		1,517	18.6	8.4
Missing		4,130	73.4	12.9
Use of over-the-counter drugs during pregnancy	Cycle 1			
Yes		676	7.6	10.8
No		1,583	19.0	8.9
Missing		4,129	73.4	12.9
Breastfeeding	Cycle 1 and 2			
Yes–was breastfed		1,884	22.7	8.9
No–never breastfed		589	6.7	11.0
Missing		3,915	70.6	13.1
Child injured	4–5 years			
Yes		626	9.2	14.2
No		5,762	90.8	11.8
Child has a condition that prevents/limits participation	4–5 years			
Yes		167	3.2	20.9
No		6,221	96.8	11.7
Child’s development (PPVT score)	4–5 years			
Delayed development		2,433	45.0	12.0
Normal development		3,955	54.7	12.0
Stressful event (ever)	Birth-5 years			
Yes		2,488	40.5	15.4
No		3,900	59.5	9.7
Death in the family	Birth-5 years			
Yes		701	11.4	16.6
No		5,687	88.7	11.4
Mother in youngest age group at birth	Cycle 1			
Mother <25 years at birth		1,386	17.9	14.7
Mother ≥25 years at birth		5,002	82.1	11.4
Mother in oldest age group at birth	Cycle 1			
Mother <40 years at birth		146	3.6	11.7
Mother ≥40 years at birth		6,242	96.4	12.0
Single parent status	Birth-5 years			
Single parent		1,314	22.9	16.7
Lived with two parents		5,074	77.1	10.6
Mother’s volunteer status	4–5 years			
Does not volunteer		2,342	35.5	10.5
Volunteers		1,929	27.4	11.9
Missing		2,117	37.1	13.6
Participates in religious activities	4–5 years			
No		3,873	55.9	11.0
Yes		2,515	44.2	13.3
Family composition	4–5 years			
Blended		1,367	24.0	15.6
Intact		5,021	76.0	10.9
Immigrant status of mother	Birth-5 years			
Mother was immigrant		649	18.0	9.6
Mother not immigrant		5,739	82.0	12.5
Household income ever below the Low Income Cut-Off (LICO)	Birth-5 years			
Ever below LICO		2,044	35.7	13.5
Never below LICO		4,344	64.3	11.2
Mother or spouse ever unemployed	Birth-5 years			
Yes		1,695	26.5	9.8
No		4,127	65.2	12.5
Missing		566	8.3	15.2
Mother has graduated high school	Birth-5 years			
No		1,290	21.9	12.7
Yes		5,098	78.1	11.8
Mother has graduated from university or college	Birth-5 years			
No		3,968	63.2	12.3
Yes		2,420	36.8	11.5
Housing tenure	Birth-5 years			
Rented (ever)		2,198	37.8	13.8
Always owned		4,190	62.2	10.9
Current smoking status of mother	4–5 years			
Daily/occasional smoker		1,994	29.6	14.7
Non-smoker		4,394	70.4	10.8
Current binge drinking status of mother	4–5 years			
Mother reported at least one binge drink occasion		1,990	27.5	14.6
Mother did not report binge drinking		4,398	72.5	11.0
Hostile or ineffective parenting	4–5 years			
Yes		739	12.8	11.9
No		4,222	64.5	12.3
Missing		1,427	22.8	11.2
Inconsistent parenting	4–5 years			
Yes		989	16.1	9.2
No/Missing		5,399	83.9	12.5
Punitive/aversive parenting	4–5 years			
Yes		1,318	20.1	13.5
No/Missing		5,070	79.9	11.6
Child sees violence on TV	4–5 years			
Often, sometimes, seldom		3,678	56.0	12.8
Never		2,710	44.0	11.0
Child changed main childcare arrangement in past 12 months	4–5 years			
Yes		1,651	22.8	12.2
No		971	15.4	12.5
Missing		3,766	61.8	11.8
Neighbourhood cohesion	Cycles 1,3			
Low		1,297	21.9	13.2
Medium/high		4,104	60.9	11.8
Missing		3,766	17.1	11.2
Neighbourhood safety	Measured once between 0–5 years (in cycle 1)			
Low		1,299	26.0	12.1
Medium/high		5,089	74.0	12.0

The CART showed that the strongest predictor of suicidal thoughts was gender (S2 Fig). Among girls, the most predictive variable was the experience of stressful life experiences and among boys, young maternal age was most predictive. Splitting was continued to build the maximal model, splitting parent nodes until no further splits were possible that would result resulting child nodes with at least of 64 individuals (the *a priori* n_min_ value). The maximal model was manually pruned, finding nine high-risk, 30 medium risk, and 13 low-risk subgroups (S2 Fig). Table 2 shows the profiles of nine high-risk subgroups that were identified by the model.

**Table 2:** Profiles of high-risk subgroups in Classification and Regression Tree model (CART), NLSCY, Canada, 1994–2009.

High-risk subgroup	% with suicidal ideation, n	Characteristics
Subgroup 1	40.6%, n = 84	• Female • Stressful life experience reported (between 0 and 5 years) • Mother was under age 25 at birth (youngest age category) • Family member died between 0 and 5 years
Subgroup 2	39.4%, n = 74	• Female • Stressful life experience reported (between 0 and 5 years) • Mother was 25 or older at birth • No prenatal problems reported • Punitive/aversive parenting style (at 4–5 years)
Subgroup 3	30.9%, n = 83	• Female • Stressful life experience reported (between 0 and 5 years) • Mother was 25 or older at birth • No prenatal problems • No punitive/aversive parenting (at 4–5 years) • Mother did not have a college/university degree • Mother or spouse reported an activity limitation (between 0 and 5 years)
Subgroup 4	30.7%, n = 81	• Female • Stressful life experience reported (between 0 and 5 years) • Mother was 25 years or older at birth • No prenatal problems reported • No punitive/aversive parenting (at 4–5 years) • Mother did not have a college/university degree • Mother or spouse did not report an activity limitation (between 0 and 5 years) • No death in the family (between 0 and 5 years) • Lived in a rental dwelling (between 0 and 5 years)
Subgroup 5	30.4%, n = 129	• Female • Stressful life experience reported (between 0 and 5 years) • Mother under age 25 at birth • No family death • Mother reported current smoking (at 4–5 years)
Subgroup 6	30.0%, n = 131	• Female • No stressful life experience reported (between 0 and 5 years) • Single parent status (between 0 and 5 years) • Exposed to violent TV (at 4–5 years)
Subgroup 7	27.7%, n = 121	• Female • No stressful life experience reported (between 0 and 5 years) • No single parent status (between 0 and 5 years) • Mother reports current smoking (at 4–5 years) • Consistent parenting (at 4–5 years) • Did not participate in religious activities (at 4–5 years)
Subgroup 8	26.6%, n = 86	• Female • Stressful life experience reported (between 0 and 5 years) • Mother was 25 or older at birth • Prenatal problems • No punitive/aversive parenting (at 4–5 years)
Subgroup 9	25.3%, n = 97	• Male • Child did not have activity limitations (at age 4–5 years) • Blended family • Mother reported current binge drinking (at 4–5 years) • Participated in religious services (at 4–5 years)

The strongest predictor of suicidal thoughts was gender. Among girls, the most predictive variable was the experience of a stressful life event while among boys, parental age was the most significant predictors. Family composition, including single parent status or blended family composition were also important predictors of high-risk. A number of factors in the pre- and early post-natal period were important predictors, including exposure to parental smoking, prenatal medical problems, prenatal use of over-the-counter medication, and lack of breastfeeding. The sensitivity of the model was 22·7% (95% CI: 19·4,26·1), specificity was 89·2% (95% CI: 88·4,90·0), positive predictive value 17·8% (95% CI: 15·1,20·5), and negative predictive value 91·8% (95% CI: 91·1,92·6). Of the original predictors, fourteen variables were included in the final logistic regression model, including gender, prenatal smoking, single parent status, serious childhood illness and others (S10 Table). The sensitivity of the logistic regression model was 9·1% (95% CI: 7·1,11·4) and specificity was 96·6% (95% CI: 96·1,97·1). Utilizing the risk grouping generated in the NLSCY-based CART model, comparable groups were created in ALSPAC. The sensitivity of the same CART model in ALSPAC was 19·9% (95% CI: 16·9,22·8), specificity was 88·0% (95% CI: 87·0,89·0), positive predictive value was 22·5% (95% CI: 19·2,25·8), and negative predictive value was 86·3% (95% CI: 85·2,87·3).

Prevalence estimates of adverse adolescent outcomes in the NLSCY ranged from 11.4% (risky health behaviours) to 26·4% (substance misuse). Suicidal thoughts were higher among all subgroups reporting one of the secondary adverse outcomes. The model built to predict suicidal thoughts was applied to the secondary outcomes in the NLSCY. Sensitivity was found to range from 29·8% (95% CI: 26·1,33·5) for antisocial behaviour to 83·8% (95% CI: 80·9,86·8) for risky health behaviours (Table 3).

**Table 3:** Sensitivity, specificity, positive predictive value, negative predictive value for secondary outcomes for high-risk classification, NLSCY, Canada, 1994–2009.

	Proportion (%)	95% CI
Antisocial behaviour		
Sensitivity	29.8	26.1,33.5
Specificity	76.9	75.8,78.0
Positive Predictive Value	11.7	10.1,13.3
Negative Predictive Value	91.4	90.7,92.2
Substance misuse		
Sensitivity	64.5	60.6,68.3
Specificity	76.2	75.1,77.3
Positive Predictive Value	13.3	11.6,15.0
Negative Predictive Value	92	91.3,92.8
Poor physical health		
Sensitivity	75.9	72.5,79.4
Specificity	84.1	83.1,85.1
Positive Predictive Value	13.4	11.4,15.5
Negative Predictive Value	91.5	90.8,92.3
Poor mental health		
Sensitivity	63.0	59.1,66.9
Specificity	76.0	74.9,77.1
Positive Predictive Value	13.7	12.0,15.4
Negative Predictive Value	92.2	91.4,92.9
Risky health behaviours		
Sensitivity	83.8	80.9,86.8
Specificity	89.5	88.7,90.3
Positive Predictive Value	13.6	11.1,16.2
Negative Predictive Value	91.2	90.5,92.0
Poor academic performance		
Sensitivity	69	65.3,72.7
Specificity	82.1	81.1,82.1
Positive Predictive Value	15.1	13.1,17.1
Negative Predictive Value	92.1	91.3,92.8

## Discussion

This large prospective study of 6,388 Canadian children showed that there are groups of related risk factors present in early life that can predict higher risk of suicidality in adolescence. While the sensitivity of the model was relatively low, it was able to identify groups of high-risk children at least seven years prior to the emergence of suicidal thoughts. Further, the sensitivity of the CART model showed a marked improvement over logistic regression (22.7% and 9.1% respectively). This model was validated using a large cohort of British children and showed similar sensitivity and specificity. Notably, we found that the early life factors linked to suicidal thoughts were also predictive of a range of adverse outcomes in adolescence including antisocial behaviour, substance misuse, poor physical health, poor mental health, risky health behaviours, and poor academic performance.

The range of factors in early life that were the most predictive were primarily markers of adversity or signaled the experience of stress during early childhood. There were some unexpected results, including one subgroup that did not experience many of the prominent early-life risk factors. In this group, the only risk factors were maternal smoking and non-participation in religious activities. For the other high risk subgroups, measures of prenatal, postnatal, and early childhood adversity were found to increase risk of later suicidal thoughts. Consistent with other epidemiological research [31,32], this study showed a relationship between the experience of early life adversity and the development of mental health problems, including suicidality. Childhood adversity, trauma, and exposure to negative conditions; including low socioeconomic status, parental mental and physical health difficulties; child maltreatment; and more, have been linked to a range of psychological difficulties including depression [33,34], anxiety disorders [33,35], and suicidality [33,35,36]. Specifically, factors in the pre- and post-natal period, including exposure to parental smoking or absence of breastfeeding were important predictors of increased risk of suicidal thoughts. This finding was consistent with the Barker’s Developmental Origins of Health and Disease hypothesis, which showed the enduring influence of factors present in the prenatal and early-life period can alter disease risk throughout the life course [37,38].

This research showed that characteristics of the high-risk subgroups show the effect of risk clustering and risk accumulation through exposure to multiple concurrent and sequential risk factors. While past research has shown that in isolation, individual risk factors do not have a strong explanatory influence on the risk of suicidal thoughts, exposure to multiple risk factor have been shown to elevate risk [39].

While it is common for youth to experience some adversity in their lifetime, youth that experience a high number of adversities may be at increased risk for psychiatric problems [40]. Additionally, many risk factors have a high level of co-occurrence, where they tend to cluster together such that the experience of one risk factor increases the chances of additional risk factors [41]. Clustered risk factors increase the likelihood of depression, psychological distress, and other mental health problems [41]. The evidence for clustering of risk factors was particularly strong when socioeconomic status was considered. Risk factors, such as childhood adversity, are distributed unequally across gender, income, race, parental education levels, and experiences of social disadvantage [34]. Youth with certain sociodemographic characteristics were more likely to report multiple adversities in a “constellation of stressors” when compared to other youth [40]. Socioeconomic variation in the risk of mental health problems, including suicidality, may be partially explained by the differential clustering of risk factors by socioeconomic status [42].

When looking across the life course, experiencing multiple risk factors may be due to the accumulation of risk, where the experience of adversity in early life increases the likelihood of exposure to subsequent stressors [34]. For example, research has shown that those who have experienced abuse in childhood are more likely to experience violence as adults [34]. In this research, we demonstrated that high-risk subgroups experienced multiple negative exposures, and it appears that the accumulation of these stressors increases the later risk of suicidal thoughts.

Further, prior research has suggested that the pathogenic effects of negative exposures early in life may effect a range of negative health and social outcomes [43]. By applying the predictive models to other negative outcomes, this study found that the models performed with better sensitivity across the range of adverse outcomes, indicating that the distal factors used to predict suicidal thoughts are more global risk factors that predict a range of adverse outcomes. The multifinality of risk factors suggests that a group of risk factors may lead to multiple adverse outcomes. The identified subgroups are typified by difficult early life conditions, which has implications for a range of outcomes in adolescence [37].

Several limitations should be noted in this study. First, this cohort experienced losses to follow-up. 48% of participants in the NLSCY between the ages of 0 and five at the start of the study did not respond to the suicide module in adolescence and were excluded. As dropout was more common among those from poorer socioeconomic conditions, this created a higher degree of homogeneity in the cohort. In the context of tree-based modeling, the systematic drop-out of individuals with shared characteristics may mean that our model may be missing branches, characterised by different risk factors. While this impacts the generalizability of the models, we do not anticipate that this would have had a dramatic influence on the observed relationships. We anticipate this will bias estimates towards the null and that our estimates are conservative. This is supported by research which indicates that cohort studies which experience selective drop-out are likely to underestimate the prevalence of psychiatric disorders [44–46]. Further, as numerous predictive variables were considered, participants were not dropped from analysis due to item non-response. For variables that had high degrees of missingness, this may have introduced heterogeneity into the subgroups. A limitation of this exploratory analytic approach was that many comparisons are conducted in the creation of CART models, which increases the risk of type one errors. While it is possible that some anomalous findings were real, it is also possible that some of the hazardous factors that appear protective in the CART model may be due to type one errors. A limitation of the validation stage was that the exact measures were not available on both the NLSCY and ALSPAC surveys. While every effort was made to select the closest proxy, there were several variables for which there was not an appropriate substitute.

These limitations are offset by the following strengths. This study was based on a large, nationally representative cohort that was followed prospectively over 14 years, and results were validated in a population-based prospective cohort from another country. The longitudinal nature of the data, long duration of follow-up, and the inclusion of somewhat rare outcomes allowed us to disentangle questions of temporality, as all risk factors proceeded the outcome. We were able to include predictive variables that are relatively rare and temporally distant to the outcomes.

## Conclusions

This research demonstrated that a range of factors in early life were able to predict negative health and social outcomes in adolescence, contributing to the growing recognition that health problems, including suicidal thoughts and other negative outcomes have shared roots in the early childhood period. This comprehensive approach is in line with the multidimensional view of health, and supports health promotion and prevention interventions that target subgroups at increased risk of many negative adolescent outcomes. Through the identification of high-risk subgroups characterized by the absence of nurturing early childhood environments, targeted health promotion programs that could be developed to intervene during the middle childhood years with the aim of attenuating risk and building resiliency in identified high-risk groups between the early life period and adolescence.

## Supporting information

S1 Table Antisocial, externalizing behaviours questionnaire and coding, NLSCY. (DOCX)

S2 Table Substance misuse questionnaire and coding, NLSCY. (DOCX)

S3 Table Poor physical health questionnaire and coding, NLSCY. (DOCX)

S4 Table Depression scale (CES-D) questionnaire and coding, NLSCY. (DOCX)

S5 Table Anxiety and Emotional Disorders (AED) questionnaire and coding, NLSCY. (DOCX)

S6 Table Risky health behaviours questionnaire and coding, NLSCY. (DOCX)

S7 Table Poor academic outcomes questionnaire and coding, NLSCY. (DOCX)

S8 Table NLSCY predictor variables. (DOCX)

S9 Table ALSPAC predictor variables. (DOCX)

S10 Table Odds ratio estimates and 95% confidence limits for stepwise selection logistic regression model, NLSCY. (DOCX)

S1 Fig Conceptual model–social ecological model of suicidality. (TIF)

S2 Fig Classification and regression tree (CART) model, NLSCY, N (unweighted) and rate of suicidal thoughts (SI). (TIF)
