# Supplementary material for: Early life predictors of adolescent suicidal thoughts and adverse outcomes in two population-based cohort studies
Source: PLoS One. 2017 Aug 10;12(8):e0183182. doi: 10.1371/journal.pone.0183182 (PMC5552309; doi:10.1371/journal.pone.0183182)
Supplement: S1 Table — (DOCX) [file pone.0183182.s001.docx]

**S1 Table. Antisocial, externalizing behaviours questionnaire and coding, NLSCY**

| **Question: “In the past 12 months, about how many times…”** | **Possible responses:** | **Coding** |
| --- | --- | --- |
| Were you questioned by the police about anything they thought you did? | 1=never  2=once or twice  3=three – four times  4=five times or more | Yes (1) = three or four times; five times or more |
| Have you run away from home? | 1=never  2=once or twice  3=three – four times  4=five times or more | Yes (1) = three or four times; five times or more |
| Have you stayed out all night without permission? | 1=never  2=once or twice  3=three – four times  4=five times or more | Yes (1) = three or four times; five times or more |
| Have you intentionally damaged or destroyed anything that didn’t belong to you? | 1=never  2=once or twice  3=three – four times  4=five times or more | Yes (1) = three or four times; five times or more |
| Have you fought with someone to the point where they needed to care for their injuries? | 1=never  2=once or twice  3=three – four times  4=five times or more | Yes (1) = once or twice; three or four times; five times or more |
| Have you carried a weapon for the purpose of defending yourself or using it in a fight? | 1=never  2=once or twice  3=three – four times  4=five times or more | Yes (1) = once or twice; three or four times; five times or more |
| Have you sold any drugs? | 1=never  2=once or twice  3=three – four times  4=five times or more | Yes (1) = once or twice; three or four times; five times or more |
| Have you attempted to touch anyone in a sexual way while knowing that they would probably object to this? | 1=never  2=once or twice  3=three – four times  4=five times or more | Yes (1) = once or twice; three or four times; five times or more |
| In the past 12 months, were you part of a gang that broke the law by stealing, hurting someone, damaging property, etc.? | 1=yes  2=no | Yes (1) = yes |
