# Supplementary material for: Early life predictors of adolescent suicidal thoughts and adverse outcomes in two population-based cohort studies
Source: PLoS One. 2017 Aug 10;12(8):e0183182. doi: 10.1371/journal.pone.0183182 (PMC5552309; doi:10.1371/journal.pone.0183182)
Supplement: S2 Table — (DOCX) [file pone.0183182.s002.docx]

**S2 Table. Substance misuse questionnaire and coding, NLSCY**

| **Question:** | **Possible responses:** | **Coding** |
| --- | --- | --- |
| Cigarettes | 1=I have never smoked  2=Only a few puffs  3=I do not smoke anymore  4=A few times a year  5=About once or twice a month  6=About 1-2 days a week  7=About 3-4 days a week  8=About 6-7 days a week | Yes (1)=About 1-2 days a week; about 3-5 days a week; about 6-7 days a week |
| Alcohol | 1=I have never drank  2=Only a few sips  3=I have only tried 1 or 2 (at least 1 drink)  4=I don’t drink anymore  5=A few times a year  6=About once or twice a month  7=About 1-2 days a week  8=About 3-4 days a week  9=About 6-7 days a week | Yes (1)=About 1-2 days a week; about 3-5 days a week; about 6-7 days a week |
| Marijuana | 1=I have never smoked  2=Not in the past 12 months  3=A few times  4=Once or twice a month  5=About 1-2 days a week  6=About 3-4 days a week  7=About 6-7 days a week | Yes (1)=Once or twice a month; 1-2 days a week; 3-5 days a week; 6-7 days a week |
| Hallucinogens | 1=Never  2=I have done it, but not in the past 12 months  3=In the past 12 months, I have used it 1-2 times  4=In the past 12 months, I have used it 3-5 times  5=In the past 12 months, I have used it 6-9 times  6=In the past 12 months, I have used it 10 times or more | Yes (1)= I have done it, but not in the past 12 months; In the past 12 months, I have used it 1-2 times; 3-5 times; 6-9 times; 10 times or more |
| Glue | 1=Never  2=I have done it, but not in the past 12 months  3=In the past 12 months, I have used it 1-2 times  4=In the past 12 months, I have used it 3-5 times  5=In the past 12 months, I have used it 6-9 times  6=In the past 12 months, I have used it 10 times or more | Yes (1)= I have done it, but not in the past 12 months; In the past 12 months, I have used it 1-2 times; 3-5 times; 6-9 times; 10 times or more |
| Prescription drugs (without a prescription) | 1=Never  2=I have done it, but not in the past 12 months  3=In the past 12 months, I have used it 1-2 times  4=In the past 12 months, I have used it 3-5 times  5=In the past 12 months, I have used it 6-9 times  6=In the past 12 months, I have used it 10 times or more | Yes (1)= I have done it, but not in the past 12 months; In the past 12 months, I have used it 1-2 times; 3-5 times; 6-9 times; 10 times or more |
| Other drugs like ecstasy, crack, cocaine, heroin, speed, etc. | 1=Never  2=I have done it, but not in the past 12 months  3=In the past 12 months, I have used it 1-2 times  4=In the past 12 months, I have used it 3-5 times  5=In the past 12 months, I have used it 6-9 times  6=In the past 12 months, I have used it 10 times or more | Yes (1)= I have done it, but not in the past 12 months; In the past 12 months, I have used it 1-2 times; 3-5 times; 6-9 times; 10 times or more |
