# Supplementary material for: Early life predictors of adolescent suicidal thoughts and adverse outcomes in two population-based cohort studies
Source: PLoS One. 2017 Aug 10;12(8):e0183182. doi: 10.1371/journal.pone.0183182 (PMC5552309; doi:10.1371/journal.pone.0183182)
Supplement: S3 Table — (DOCX) [file pone.0183182.s003.docx]

**S3 Table. Poor physical health questionnaire and coding, NLSCY**

| **Question:** | **Possible responses:** | **Coding** |
| --- | --- | --- |
| Poor general health | 1=Excellent  2=Very good  3=Good  4=Fair  5=Poor | Yes (1)=Fair; Poor |
| In the past 6 months, how often have you had the following: Headache | 1=Seldom/never  2=About once a month  3=About once a week  4=More than once a week  5=Most days | Yes (1)=Most days |
| In the past 6 months, how often have you had the following: Stomachache | 1=Seldom/never  2=About once a month  3=About once a week  4=More than once a week  5=Most days | Yes (1)=Most days |
| In the past 6 months, how often have you had the following: Backache | 1=Seldom/never  2=About once a month  3=About once a week  4=More than once a week  5=Most days | Yes (1)=Most days |
