# Supplementary material for: Early life predictors of adolescent suicidal thoughts and adverse outcomes in two population-based cohort studies
Source: PLoS One. 2017 Aug 10;12(8):e0183182. doi: 10.1371/journal.pone.0183182 (PMC5552309; doi:10.1371/journal.pone.0183182)
Supplement: S4 Table — (DOCX) [file pone.0183182.s004.docx]

**S4 Table. Depression scale (CES-D) questionnaire and coding, NLSCY**

| **Question:** | **Possible responses:** | **Coding** |
| --- | --- | --- |
| I felt that I could not shake off the blues even with help from my family or friends | 1=Rarely/ none of the time (less than one day)  2=  3=  4=most or all of the time (5-7 days) | Continuous scores range from 0 to 36.  1= depressed (9-36 score)  2= not depressed (0-8 score) |
| I had trouble keeping my mind on what I was doing |  |  |
| I felt depressed |  |  |
| I felt that everything I did was an effort |  |  |
| I felt hopeful about the future |  |  |
| My sleep was restless |  |  |
| I was happy |  |  |
| I felt lonely |  |  |
| I enjoyed life |  |  |
| I had crying spells |  |  |
| I felt that people disliked me |  |  |
