# Supplementary material for: Early life predictors of adolescent suicidal thoughts and adverse outcomes in two population-based cohort studies
Source: PLoS One. 2017 Aug 10;12(8):e0183182. doi: 10.1371/journal.pone.0183182 (PMC5552309; doi:10.1371/journal.pone.0183182)
Supplement: S5 Table — (DOCX) [file pone.0183182.s005.docx]

**S5 Table. Anxiety and Emotional Disorders (AED) questionnaire and coding, NLSCY**

| **Question:** | **Possible responses:** | **Coding** |
| --- | --- | --- |
| I am unhappy or sad | 1=Never true/ not true  2=Sometimes/ somewhat true  3=Often/very true | Continuous scores range from 0 (never/rarely true) on all symptoms to 14 (often/very true) on all symptoms.  1= medium/high anxiety (8-14 score)  2= low anxiety (0-14 score) |
| I am not as happy as other people my age |  |  |
| I am too fearful or nervous |  |  |
| I worry a lot |  |  |
| I am nervous, high strung, or tense |  |  |
| I have trouble enjoying myself |  |  |
| I cry a lot |  |  |
