# Supplementary material for: Early life predictors of adolescent suicidal thoughts and adverse outcomes in two population-based cohort studies
Source: PLoS One. 2017 Aug 10;12(8):e0183182. doi: 10.1371/journal.pone.0183182 (PMC5552309; doi:10.1371/journal.pone.0183182)
Supplement: S6 Table — (DOCX) [file pone.0183182.s006.docx]

**S6 Table. Risky health behaviours questionnaire and coding, NLSCY**

| **Question:** | **Possible responses:** | **Coding** |
| --- | --- | --- |
| Operated a motor vehicle (car, motorcycle, boat) after drinking or doing drugs (asked of 16-17 year olds) | 1=Never  2=Once or twice  3=3-4 times  4=5 times or more | Yes (1)=3-4 times; 5 times or more |
| Been a passenger in a vehicle after the driver drank or used drugs (asked of 16-17 year olds) | 1=Never  2=Once or twice  3=3-4 times  4=5 times or more | Yes (1)=3-4 times; 5 times or more |
| Use a seat belt when riding in a car | 1=Always  2=Often  3=Sometimes  4=Seldom or never  5=Usually there is not seatbelt where I sit | Yes (1)=Seldom or never |
| Sex – no protection (16-19 year olds) (look at wording) | 1=Yes  2=No | Yes (1)=Yes |
| Sex – no condom (14-15 year olds) | 1=Yes  2=No | Yes (1)=If NO (2) to condom AND NO (2) to other forms of birth control |
| Did you or your partner use other methods of birth control (birth control pills, diaphragm, etc.) the last time you had consensual sexual intercourse (Asked of 14-15 year olds) | 1=Yes  2=No |  |
