# Supplementary material for: Early life predictors of adolescent suicidal thoughts and adverse outcomes in two population-based cohort studies
Source: PLoS One. 2017 Aug 10;12(8):e0183182. doi: 10.1371/journal.pone.0183182 (PMC5552309; doi:10.1371/journal.pone.0183182)
Supplement: S7 Table — (DOCX) [file pone.0183182.s007.docx]

**S7 Table. Poor academic outcomes questionnaire and coding, NLSCY**

| **Question:** | **Possible responses:** | **Coding** |
| --- | --- | --- |
| How well do you think you are doing in your school work? | 1=Very well  2=Well  3=Average  4=Poorly  5=Very poorly | Yes (1)=Poorly; Very poorly |
| Skipped a day of school without permission | 1=Never  2=Once or twice  3=3-4 times  4=5 times or more | Yes (1)=3-4 times; 5 times or more |
| Been suspended from school | 1=Never  2=Once or twice  3=3-4 times  4=5 times or more | Yes (1)=Once or twice; 3-4 times; 5 times or more |
| Dropped out of school (asked of 14-15 year olds) | 1=Never  2=Once or twice  3=3-4 times  4=5 times or more | Yes (1)=Once or twice; 3-4 times; 5 times or more |
