# Supplementary material for: Early life predictors of adolescent suicidal thoughts and adverse outcomes in two population-based cohort studies
Source: PLoS One. 2017 Aug 10;12(8):e0183182. doi: 10.1371/journal.pone.0183182 (PMC5552309; doi:10.1371/journal.pone.0183182)
Supplement: S8 Table — (DOCX) [file pone.0183182.s008.docx]

**S8 Table. NLSCY predictor variables**

| VARIABLE | QUESTION |
| --- | --- |
| Presence of prenatal medical problems | The PMK (person most knowledgeable, in most cases, mother) was asked if the mother of the study child had medical issues during the prenatal period, including diabetes, high blood pressure, and other physical problems. |
| Prenatal smoking | The PMK was asked if the mother of the study child had smoked during stage 1, stage 2, or stage 3 of pregnancy. |
| Prenatal alcohol drinking | PMKs were asked “How frequently did [you/the mother] consume alcohol during your pregnancy with [the study child]?” |
| Prenatal prescription drug use | Mothers were asked “Did you take any prescription medications during your pregnancy with [the study child]?” |
| Prenatal over-the-counter drug use | Mothers were asked if they took over-the-counter drugs during pregnancy. |
| Low birth weight | Mothers were asked to report the weight of the study child at birth. |
| Prematurity |  |
| Infant special medical care after birth | Mothers were asked if their child received special medical care after birth. |
| Maternal hospitalization after birth | Mothers were asked “Were/was you/her/his mother hospitalized for special medical care for any period immediately following the birth of [the study child]?” |
| Post-partum depression | PMKs were asked “After ___’s delivery, did you/her/his mother suffer from postpartum depression” |
| Breastfeeding | In order to assess breastfeeding, responses to two questions were combined. Mothers were asked if they were currently breastfeeding. Of those not currently breastfeeding, they were asked “did you/her his mother breast-feed him/her even if only for a short time” were also coded as ‘yes.’ |
| Gender | Gender of the child was asked of the PMK |
| Racial background/ ethnicity | Racial background/ethnicity was determined by a series of questions, which asked the PMK to “best describe [the child’s] race/colour.” For this analysis, two predictors were included in the analysis. The first variable approximates visible minority status, using the question which asked if “White” was the how the PMK would best describe the child’s race/colour. Similarly, in order to estimate Aboriginal status, individuals who responded that “Native/Aboriginal people (including North American Indian, Métis, Inuit/Eskimo)” best described their race/colour” |
| Immigration | The PMK was asked to indicate if the child was an immigrant. |
| Rural or urban residence (at age 4-5) | The PMK was asked to indicate if the child was currently living in a rural or urban area. |
| Child’s general health (at age 4-5) | Parents/PMKs were asked to report on the general health of their child at age 4-5 by the following question: “In general, would you say…’s health is: excellent, very good, good, fair, poor, don’t know, refusal.” |
| Physical activity (at age 4-5) | PMKs were asked to report on their child’s physical activity compared to other children at age 4-5. |
| Chronic conditions | The PMK was asked a series of questions regarding the presence of chronic conditions including diabetes, heart disease, and epilepsy. |
| Pain and discomfort (at age 4-5) | The PMK was asked if the child at age 4-5 is usually free of pain and discomfort. |
| Injuries (at age 4-5) | PMKs were asked if the child had been injured in the past 12 months. |
| Activity limitation (at age 4-5) | PMKs were asked to indicate how many activities are prevented due to pain or discomfort. |
| Activities | PMKs were asked a series of questions about the child’s participation in various activities, including participation in early childhood activities, playing sports with and without a coach, participation in gymnastics or dance, arts, drama, or music, or guides, scouts and/or other clubs. |
| Hyperactivity/ Inattention (at age 4-5) (Behaviour subscale) | Symptoms of internalizing problems, including hyperactivity, inattention, and impulsive behaviours were estimated using an 8 question subscale, with possible scores ranging form 0-16. Higher scores indicated the presence of more symptoms of hyperactivity. |
| Conduct problems (at age 4-5) (Behaviour subscale) | Symptoms of externalizing behaviour were estimated using a subscale consisting of 6 questions, with possible score ranging from 0-12. |
| Prosocial behaviours (at age 4-5) (Behaviour subscale) | Presence of prosocial behaviours was estimated using the prosocial behaviour subscale, with possible scores ranging from 0-20, where a 0 indicated the absence of prosocial behaviour. |
| Indirect aggression (at age 4-5) (Behaviour subscale) | Indirect aggression was estimated using subscale with possible scores ranging from 0-10. |
| Emotional disorders/ anxiety subscale (at age 4-5) (Behaviour subscale) | Emotional disorders/anxiety was assessed using an 8-question subscale with possibly scores ranging from 0-16, where a higher score reflected more symptoms of anxiety. |
| The Peabody Picture Vocabulary Test – Revised (PPVT-R) standardized score | The Peabody Picture Vocabulary Test – Revised (PPVT-R), developed by Llyod and Leota Dunn, was used to measure school readiness and cognitive ability for children in the 4-5 age group. The test was administered to the child in the home and the interviewer read out words and the child was to look at pictures on an easel and identify the picture that matched the word read out by the interviewer. The raw score was calculated then standardized, taking the child’s age into account using 2-month age groups. The score was adjusted so that the mean of standard scores was 100 and the standard deviation was 15 for all age groupings. |
| Stressful life experiences | PMKs were asked to report if their child had experienced any event that cause a great deal of stress or worry between birth and age 5. |
| Exposure to intimate partner violence | PMKs were asked to report if their child saw adults fighting or hitting. |
| Exposure to violence on TV | PMKs were asked to report if their child saw violence on TV. |
| Teenage status of parents at birth | The NLSCY asked the age of the biological parents at birth. |
| Age group of PMK at birth | Two dichotomous variables were created to capture the age of mother at birth. |
| Low income (SES) | Socioeconomic status was measured as the ratio of the household income to the low income cut off score (LICO), established by Statistics Canada. The LICO indicates an income level at which a family is likely to spend a large portion of their income on necessities, accounting for family size and the community they live in. |
| Education level of PMK | The education level of the PMK was considered as a potential predictor of suicidal ideation. On the NLSCY, PMKs are asked about their highest level of education obtained, with four possible responses: less than secondary, secondary school graduation; beyond secondary school graduation; university or college degree. |
| Unemployment of PMK or spouse | The PMK and spouse were asked about their work status in the past year. |
| Housing tenure (rented vs. owned) | PMKs were asked “Is this dwelling owned by a member of this household (even if being paid for)”. |
| Immigrant status of PMK | In order to determine if PMKs were immigrants, the response to ‘number of years since first moving to Canada’ was considered. |
| Religion | PMK’s were asked “what, if any, is your/…’s religion?.” |
| Participation in religious activities | Of those who reported a religion, PMK’s were asked the following question: “Other than on special occasions (such as weddings, funerals or baptisms), how often did ... attend religious services or meetings in the past 12 months?” |
| Single parent status | Child’s single parent status was assessed during cycles 1-3. |
| Intact vs. blended family | Family composition was assessed using the blended family variable. |
| Poor parental general health | PMKs were asked to report on their general health when their child was ages 4-5 by the following question: “In general, would you say…’s health is: excellent, very good, good, fair, poor, don’t know, refusal.” |
| Presence of chronic conditions (PMK or spouse) | PMKs and their spouses/partners were asked if they had chronic conditions. |
| Parental activity limitation (PMK or spouse) | PMKs and their spouses/partners were also asked if they had a condition that limited their activity. |
| Parental smoking | PMKS were asked about their smoking habits. |
| Parental binge drinking | PMKs were asked about their current alcohol consumption, including the frequency of binge drinking (i.e., 5 or more drinks on one occasion). |
| Maternal depression score | Maternal depression was measured using the 12-item version of the National Institute of Mental Health’s Centre for Epidemiological Studies Depression scale (CES-D) developed by L.S. Radloff in 1977. The 12-item version of the scale was developed by Dr. M. Boyle of the Chedoke-McMaster Hospital of McMaster University and was rescaled to produce a cut-off proportionate to the full, 20-item CES-D. The modified CES-D showed good internal consistency and had a Cronbach’s alpha of the scale was 0.82. |
| Positive interactions parenting style | Parenting characterized by positive interactions were assessed in the NLSCY by a 5 item scale with a possible scores ranging from 0-20, where a high score indicated a high number of positive interactions with the child. Questions included “How often do you and [study child] laugh together?”. This scale has been shown to have good concurrent validity, and an internal-consistency reliability of 0.81. |
| Hostile or ineffective parenting style | Hostile or ineffective parenting style was assessed using a 7 question scale which included questions such as “How often do you get annoyed with [study child] for saying or doing something s/he is not supposed to?”, “How often do you get angry when you punish [study child]?”, “How often do you think that the kind of punishment you give him/her depends on your mood?”, and “How often do you feel you are having problems managing him/her in general?”. Each question had 5 response options ranging from ‘never’ to ‘all the time’, with a total score ranging between 0 and 28, with a high score indicating a high degree of hostile/ineffective parenting. |
| Inconsistent parenting style | There were five items that comprise the measurement of parental consistency including “When you give your child a command, what proportion of the time so you make sure that he/she does it?” and “When your child breaks the rules or does things that he/she is not supposed to, how often do you ignore it, do nothing?”. The total score ranged between 0-20, and those with a low score were considered to have an inconsistent parenting style. |
| Punitive or aversive parenting style | There were four items that comprise the measurement of punitive or aversive parenting style. The total score ranged between 0-19, and those with a high score were considered to have a punitive or aversive parenting style. |
| Family functioning score | Family functioning was measured using a 12-item scale that provides a global assessment of family functioning and a measure of the quality of relationships between family measures, including problem solving, communication, roles, affective involvement and responsiveness, and behaviour control. The PMK or the spouse of the PMK answered the family functioning questions as part of the Parent Questionnaire. Family functioning scores ranged from 0-36, where higher scores indicated higher levels of family dysfunction. |
| Social support score | Perceived social support was measured by a shortened version of the Social Provisions Scale, originally developed by Dr. Carolyn Cutrona and Dr. Daniel Russell of Iowa State University. The shortened version was developed to measure guidance, reliable alliances, and attachments in the context of social relationships in order to determine the level of support received from family, friends, and others. Parents (either the PMK or the spouse/partner) answered social support questions as part of the Parent Questionnaire, with a total score ranging between 0-18, where a high score indicated high levels of social support. |
| Changes in childcare arrangements | PMKs were asked to “Overall, how many changes in child care arrangements has your child experienced since you began using child care, excluding periods of care by yourself (or spouse/partner)?”. |
| Social support score | The social support score is derived using 8 items to get a total score between 0 and 24, where a high score indicates a high level of social support. |
| Parental participation in volunteering | PMKs were asked if they participated in a volunteer activity in the past 12 months. |
| Social skills index (teacher rated) | Teachers of study children were administered a questionnaire and were asked to gate the level of social skills the children had. The social skills score ranged between 0 and 40, with a low score indicating poor social skills. |
| Neighbourhood cohesion scale (Neighbours score) | The neighbourhood cohesion scale was developed to measure the social utility of a neighbourhood. The PMK or their spouse/partner was asked to report on the level of cohesion in the neighbourhood, including how willing neighbours are to help each other, deal with local problems, keep an eye out for trouble, watch out for the safety of neighbourhood children, and be a role model for children. 0-15, high score indicating a high degree of neighbor cohesiveness. |
| Neighbourhood safety scale | In the NLSCY, perceived neighbourhood safety was measured in cycle 1 using the Neighbourhood Safety Scale, which measured the extent to which the PMK or their spouse/partner felt that there was a sense of safety in the neighbourhood. |
