# Supplementary material for: Early life predictors of adolescent suicidal thoughts and adverse outcomes in two population-based cohort studies
Source: PLoS One. 2017 Aug 10;12(8):e0183182. doi: 10.1371/journal.pone.0183182 (PMC5552309; doi:10.1371/journal.pone.0183182)
Supplement: S9 Table — (DOCX) [file pone.0183182.s009.docx]

**S9 Table. ALSPAC predictor variables**

| **VARIABLE** | **QUESTION** |
| --- | --- |
| **Prenatal over-the-counter medication use** | Mothers were asked at 18 weeks gestation about a list of pills, ointments, and medications. Mothers were asked about a variety of conditions, several of which are likely to be treated using over-the-counter medications, including nausea, heartburn, vomiting, and migraines. A derived variable was generated combining the responses to use of a medication for nausea, heartburn, vomiting, and migraines. |
| **Prenatal smoking** | Mothers were asked about “how many cigarettes per day are you yourself smoking at the moment” at 32 weeks gestation. |
| **Prenatal problems** | Mothers were asked about health during gestation at multiple time points, but were specifically asked about gestational diabetes and hypertension in pregnancy at 12 weeks. Mothers were asked at 12 weeks if they have a history of diabetes. Of those with a history, they were asked if they had diabetes in pregnancy only. Similarly, all mothers were asked if they had a history of hypertension. Of those that said yes, they were asked a follow-up question if the hypertension was only during pregnancy. |
| **Breastfeeding** | Mothers were asked at 4 weeks and 6 months about breastfeeding. |
| **Gender** | Gender of the child was recorded at birth (variable kz021) |
| **Rural/urban** |  |
| **Child injury** | A series of questions about the study child injuries in the past 12 months was asked at 3 years, 2 months. |
| **Child activity limitation** | ALSPAC does not ask specifically about activity limitations of children. The closest approximation is text which details chronic conditions experienced by members of the family. As a close proxy for the NLSCY variable does not exist in the dataset, this was excluded from analysis. |
| **PPVT** | The WASI module was asked in ALSPAC, which measures IQ and developmental progress, however, an issue was discovered with the scoring of the measure so the data has not yet been released until the issues can be resolved. |
| **Conduct disorder** | Conduct disorder was one of the disorders measured in the ALSPAC at 3 years, 6 months. Each participant was given a score, ranging from 0 to 16. As per Goodman (2001; 1997), those who score at the 90^th^ percentile or above, corresponding to a score of 7 or above can be said to have a conduct disorder |
| **Experience of any stressful life experience between birth and 5 years** | The mother was asked at several time points about various stressful experiences (at 8 weeks; 2 years, 9 months; 3 years, 11 months; and 5 years, 1 month). All participants were asked about a particular stressful experience with the following response options: “yes, and it affected me a lot”, “yes, moderately affected”, “yes, mildly affected”, and “yes, but did not affect me at all” and “no, did not happen”. As a more comprehensive list of events was included in the ALSPAC module than were on the NLSCY module, only events that replicate those included in the NLSCY module were included in the summary variable. Further, as the NLSCY asks about events that caused a great deal of stress or worry for the child, only events to which the mother reported being greatly or moderately affected were included in the derived variable. The variables from the stressful included for this summary variable are as follows: your partner died, one of your children died, a friend or relative died, one of your children was ill, your partner was ill, a friend or relative was ill, you were admitted to hospital, you were divorced, you were very ill, your partner went away, you and your partner were separated, you moved house, your partner was physically cruel to you, you got married, your partner was physically cruel to your children, your partner was emotionally cruel to you, your partner was emotionally cruel to your children, you were emotionally cruel to your children. |
| **Death in the family between (birth and 5 years)** | Mothers were asked at 8 weeks; 2 years, 9 months; 3 years, 11 months; and 5 years, 1 month if “a friend or relative died”. |
| **Young maternal age at birth (under 25 years)** | In ALSPAC, maternal age (in years) was provided at 8 weeks gestation, so this time point was used to create a “young maternal age” predictor. |
| **Old maternal age at birth (40 or older)** | Maternal age (in years) was provided at 8 weeks gestation. |
| **Immigrant status of parents** | Immigrant status of the mother/partner is not specifically asked in ALSPAC, but both the mother and partner were asked to provide information about their birth, including to indicate “where parents lived at time of birth”. |
| **No maternal basic education level** | Educational attainment of the mother was ascertained in pregnancy. There are important differences between UK and Canadian education systems, which made it challenging to find the equivalent educational cut-off that is similar to achieving a high school diploma in Canada, and previous authors use various cut-off points for educational qualifications. |
| **No maternal college/university degree** |  |
| **Unemployment of mum or spouse** | Job situation of mother and partner were asked at 8 weeks. |
| **Financial difficulties** | Similar to the Low Income Cut-off measure used in the NLSCY, the financial difficulties score uses 5 questions to assess how difficult it is for the mother to afford food, clothing, heat, rent/mortgage, and things needed for the baby with a final score ranging between 0 and 15. |
| **Housing tenure** | Housing tenure was asked at 8 weeks gestation. |
| **No participation in religious activities** | At 5 years, 1 month, mothers were asked about the frequency at which she goes to a place of worship. |
| **Parental activity limitation** | ALSPAC does not ask specifically of activity limitation resulting from chronic conditions, so the closest proxy available was a single item question that asked about present health as “fit and well”. |
| **Maternal current smoking** | Mothers were asked about the number of cigarettes she currently smokes per day. |
| **Maternal binge drinking** | Mothers were asked to report on their current alcohol use each day of the week by type of alcohol consumed (beer, wine, cider, etc) at 3 years, 11 months. |
| **Single parent status** | Single parent status was assessed at multiple times throughout early childhood, including: 8 weeks; 8 months; 1 year, 9 months; 2 years, 9 months; and 3 years, 11 months |
| **Intact vs. blended family** | Family composition at 3 years, 11 months was assessed based on responses to several questions. |
| **Inconsistent parenting** | Parenting styles were measured quite differently on the NLSCY and ALSPAC. On the NLSCY, there is a parenting subscale that measures the level of consistency in parenting. The closest proxy included on ALSPAC is a single item question asked at 3 year, 6 months: "do you start by being firm then usually give way." |
| **Hostile/ineffective parenting** | Similarly, hostile/ineffective parenting was measured using a multi-item scale in the NLSCY, which asks questions about how the parent feels managing and disciplining their child. The closest approximation of this is a single item asked at 4 years, 9 months: "Who is the most frequent winner of battle of wills?" |
| **Punitive/aversive parenting** | Three questions asked at 4 years, 9 months were similar to questions asked on the NLSCY's punitive/aversive parenting style scale. These items included ‘parent tries to reason with child when child has tantrum’, ‘parent slaps or hits child when child has tantrum’, and ‘parent shouts at child when child has tantrum’. |
| **TV exposure** | A predictor in the NLSCY models was exposure to violence on TV; however, ALSPAC does not specifically ask participants about this. |
| **Change in childcare arrangements** | ALSPAC does not specifically ask about changes in childcare providers, however, there is an extensive module, which asks when about details of weekday childcare. |
| **Parental volunteering** | Mothers were asked about their community involvement at 5 years, 1 month. |
| **Low social support** | Social support was measured using a multi-item scale, and a score between 0 and 30 was derived for participants, where a high score indicates high social support and a low score indicates low social support. This module was asked at several time points during early childhood at the following ages: 8 weeks; 8 months; 1 year, 9 months. The derived social support scores available at all three time points put missing values to the mode. |
| **Low neighbourhood cohesion** | The closest proxy to the neighbourhood cohesion score included on ALSPAC is the since item that asks the mother is she “thinks the neighbourhood is a good place to live”, which is asked at 2 years 9 months and 5 years, 1 month. |
| **Low neighbourhood safety** | Neighbourhood safety is estimated from the neighbourhood stress score, which ranges from 0-23, where high scores indicate a high level of neighbourhood stress or alternately, a low level of neighbourhood safety. |
