# Supplementary material for: Early life predictors of adolescent suicidal thoughts and adverse outcomes in two population-based cohort studies
Source: PLoS One. 2017 Aug 10;12(8):e0183182. doi: 10.1371/journal.pone.0183182 (PMC5552309; doi:10.1371/journal.pone.0183182)
Supplement: S10 Table — (DOCX) [file pone.0183182.s010.docx]

**S10 Table. Odds ratio estimates and 95% confidence limits for stepwise selection logistic regression model, NLSCY**

| Effect | Point Estimate | 95% Wald Confidence Limits | |
| --- | --- | --- | --- |
| Prenatal smoking (missing vs. no) | 1.358 | 1.100 | 1.677 |
| Prenatal smoking (yes vs. no) | 1.691 | 1.249 | 2.289 |
| Gender (female vs. male) | 2.116 | 1.799 | 2.487 |
| Participation in religious activities at 4-5 years (yes vs. no) | 0.815 | 0.696 | 0.955 |
| Conduct disorder at 4-5 years (yes vs. no) | 1.311 | 1.028 | 1.672 |
| Moved between 0-5 years (yes vs. no) | 1.524 | 1.088 | 2.135 |
| Was ill between 0-5 years (yes vs. no) | 2.069 | 1.374 | 3.113 |
| Change member of the household between 0-5 years (yes vs. no) | 1.698 | 1.043 | 2.765 |
| Mother or father was a teen at birth (yes vs. no) | 1.553 | 1.045 | 2.306 |
| Ever part of a single parent family (yes vs. no) | 1.320 | 1.093 | 1.595 |
| Inconsistent parenting style (yes vs. no) | 0.726 | 0.579 | 0.910 |
| Exposed to violent TV at 4-5 years (yes vs. no) | 1.204 | 1.025 | 1.414 |
| Teacher-rated social skills at 4-5 years (missing vs. medium/high ) | 0.725 | 0.597 | 0.880 |
| Teacher-rated social skills at 4-5 years (low vs. medium/high) | 1.252 | 0.801 | 1.957 |
| Neighbourhood cohesion score (missing vs. medium/high) | 1.045 | 0.839 | 1.303 |
| Neighbourhood cohesion score (low vs. medium/high) | 1.269 | 1.049 | 1.535 |
