# Supplementary figures and images for: Early life predictors of adolescent suicidal thoughts and adverse outcomes in two population-based cohort studies
Source: PLoS One. 2017 Aug 10;12(8):e0183182. doi: 10.1371/journal.pone.0183182 (PMC5552309; doi:10.1371/journal.pone.0183182)

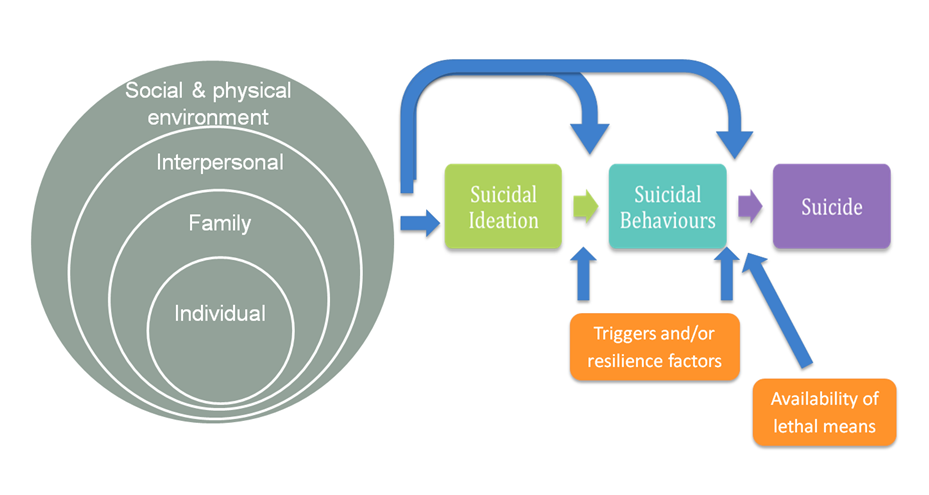

Supplement: S1 Fig — (TIF) [file pone.0183182.s011.tif]

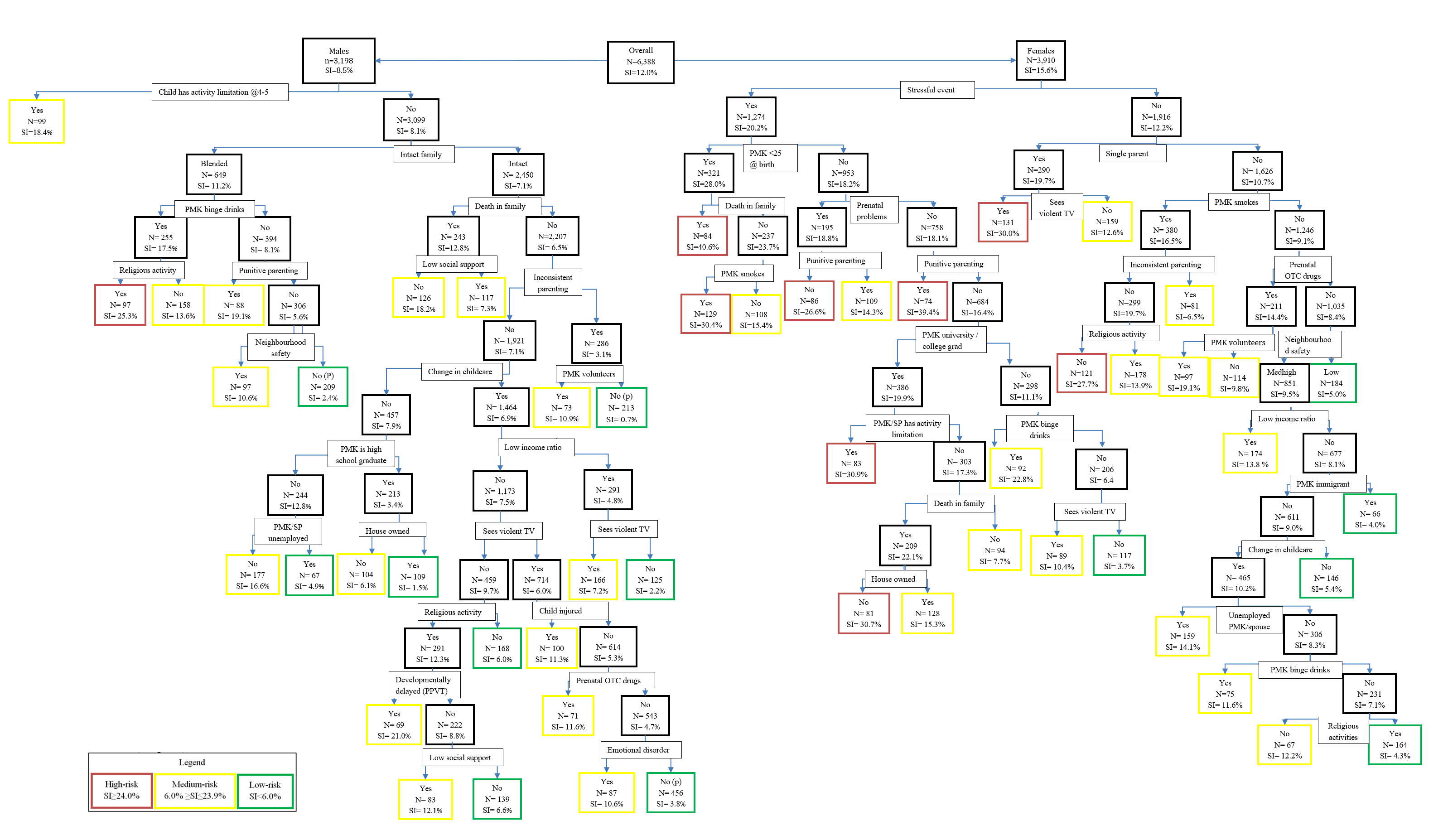

Supplement: S2 Fig — (TIF) [file pone.0183182.s012.tif]
